# Early-onset ventilator-associated pneumonia incidence in intensive care units: a surveillance-based study

**DOI:** 10.1186/1471-2334-11-236

**Published:** 2011-09-06

**Authors:** Philippe Vanhems, Thomas Bénet, Nicolas Voirin, Jean-Marie Januel, Alain Lepape, Bernard Allaouchiche, Laurent Argaud, Dominique Chassard, Claude Guérin

**Affiliations:** 1 Hospices Civils de Lyon, Infection Control Unit, Edouard Herriot Hospital, Lyon, France; 2 Laboratory of Epidemiology and Public Health, CNRS UMR 5558, University of Lyon, University of Lyon 1, Lyon, France; 3 Health Care Evaluation Unit, Institute of Social and Preventive Medicine-IUMSP, University of Lausanne, Lausanne, Switzerland; 4 Hospices Civils de Lyon, Intensive Care Unit, Lyon-South Hospital, Pierre-Bénite, France; 5 Hospices Civils de Lyon, Surgical Intensive Care Unit, Edouard Herriot Hospital, Lyon, France; 6 Hospices Civils de Lyon, Medical Intensive Care Unit, Edouard Herriot Hospital, Lyon, France; 7 Hospices Civils de Lyon, Intensive Care Unit, Mother-Infant Hospital, Bron, France; 8 Hospices Civils de Lyon, Medical Intensive Care Unit, Croix-Rousse Hospital, Lyon, France

## Abstract

**Background:**

The incidence of ventilator-associated pneumonia (VAP) within the first 48 hours of intensive care unit (ICU) stay has been poorly investigated. The objective was to estimate early-onset VAP occurrence in ICUs within 48 hours after admission.

**Methods:**

We analyzed data from prospective surveillance between 01/01/2001 and 31/12/2009 in 11 ICUs of Lyon hospitals (France). The inclusion criteria were: first ICU admission, not hospitalized before admission, invasive mechanical ventilation during first ICU day, free of antibiotics at admission, and ICU stay ≥ 48 hours. VAP was defined according to a national protocol. Its incidence was the number of events per 1,000 invasive mechanical ventilation-days. The Poisson regression model was fitted from day 2 (D2) to D8 to incident VAP to estimate the expected VAP incidence from D0 to D1 of ICU stay.

**Results:**

Totally, 367 (10.8%) of 3,387 patients in 45,760 patient-days developed VAP within the first 9 days. The predicted cumulative VAP incidence at D0 and D1 was 5.3 (2.6-9.8) and 8.3 (6.1-11.1), respectively. The predicted cumulative VAP incidence was 23.0 (20.8-25.3) at D8. The proportion of missed VAP within 48 hours from admission was 11% (9%-17%).

**Conclusions:**

Our study indicates underestimation of early-onset VAP incidence in ICUs, if only VAP occurring ≥ 48 hours are considered to be hospital-acquired. Clinicians should be encouraged to develop a strategy for early detection after ICU admission.

## Background

The epidemiological surveillance of healthcare-associated infections (HAIs) in intensive care units (ICUs) provides clinicians and caregivers with trend descriptions and contributes to HAI prevention [1-4]. When studies from such epidemiological surveillance programs are carried out, standardized definitions of risk factors for HAI must be used. However, these distinctions as well as the terminology adopted change over time. Indeed, in the last Centers for Disease Control and Prevention definition, HAI replaced the term "hospital-acquired infection" [5].

To exclude community-acquired infections, it was acknowledged that a period of 48 hours between ICU admission and the onset of symptoms was required to identify cases as hospital-acquired infections [1-4,6-10]. The time window of 48 hours was first conceived by US National Nosocomial Infection Surveillance (NNIS). The NNIS/National Healthcare Safety Network retained this value for bacterial infections because of their typical incubation period [11]. However, the statement: 1) is both conservative for some microorganisms with longer incubation periods (i.e. *Mycoplasma pneumonia*) or some viruses (i.e. influenza) and restrictive for others (i.e. *Streptococcus pneumoniae*) [12], and 2) does not take device exposure into account. In France, nosocomial infection surveillance networks adopted this time period. The main consequence is that HAIs occurring before the second day after admission are not considered.

Early-onset, ventilator-associated pneumonia (VAP), arising within 48 hours after device exposure, should be designated as HAI [5]. However, very few data support this view [13]. It has been noted that colonization of the trachea in the first 24 hours after intubation occurs frequently in head trauma patients and predicts the development of early-onset pneumonia [14]. Because no data on early-onset VAP are readily available, its incidence within 48 hours after ICU admission can be estimated from observed VAP.

Additional assessment of the incidence of very early-onset VAP is important to discuss these new definitions of hospital-acquired VAP and to draw the attention of clinicians towards early adverse events. The primary objective of the present study was to estimate the incidence of early-onset VAP in ICUs within the first 48 hours after admission.

## Methods

### Setting

Analysis was based on prospective data from 11 ICUs of Lyon hospitals in France. These ICUs participate in a national HAI surveillance network that has been described in detail elsewhere [15]. Briefly, all ICU patients staying 48 hours or more were included in the surveillance program until their ICU discharge. The data were collected prospectively over the year on a standardized collection form, which comprised demographic characteristics, the severity of underlying diseases, risk factors for HAIs, exposure to mechanical ventilation, central venous catheters, urinary catheters, date and site of infection, etiological agents, and patient outcome.

Informed consent was waived because data were extracted from the surveillance database. According to French law, a study like this one does not require ethics committee approval because it is observational and based on a surveillance database approved under national regulations (*Comité National Informatique et Liberté*).

### Inclusion criteria

Patients discharged between 01/01/2001 and 31/12/2009, hospitalized ≥ 48 hours in ICUs, were included in the present study if they met the following criteria: 1) first ICU admission, 2) not admitted from hospital, 3) free of antibiotics at the time of ICU admission, 4) neither intubated nor tracheotomized at the time of ICU admission, 5) intubated or tracheotomized during the first 24 hours after ICU admission. Patients admitted from other wards or undergoing tracheal intubation or tracheotomy or antibiotics prior to ICU admission were excluded.

### Definitions of VAP

VAP [16] was defined according to the following:

- Chest X-rays exhibiting lung infiltrates;

- Temperature > 38°C or leukocyte count > 12,000/mm^3 ^or < 4,000/mm^3^;

- One of the following: 1) sputum modification, 2) suggestive auscultation, 3) low oxyhemoglobin saturation, or 4) increased pulmonary oxygen consumption;

- And 1 of the following: 1) directed bronchoalveolar lavage (BAL)-positive culture at a threshold of 10^4 ^cfu/ml in case of BAL or 10^3 ^cfu/ml in case of non-bronchoscopic protected specimen brushing [17], or 2) fiberoptic bronchoscopy specimen-positive culture at a threshold of 10^6 ^cfu/ml, or 3) 1 of the following: positive pleural or blood cultures without any other site of infection, pulmonary or pleural abscess, histopathological evidence of pneumonia or cultures positive for specific agents.

Microbiological specimens were always obtained before the introduction of new antibiotics and as soon as possible after the identification of clinical or radiological criteria of pneumonia. In case of consecutive VAP episodes, only the first episode was considered for analysis.

### Statistical analysis

The primary end-point was the incidence of early-onset VAP during ICU stay. Therefore, the follow-up period in the present study was restricted from day 0 (D0, ICU admission) to D8 of ICU stay. Patients were followed up from admission to VAP occurrence or were censored at the end of mechanical ventilation exposure or at D8 if no VAP had transpired. The enrolled population was described. At each day of this follow-up, the observed number of incident VAP cases, the number of patients at risk for that day, and the main characteristics of patients who developed VAP were recorded. Because the exact hour of the beginning of exposure was not evident, the number of invasive mechanical ventilation days was divided by 2 to estimate mechanical ventilation days at the first and last days. The hypothesis was that, on average, patients were exposed to mechanical ventilation for 12 hours.

Categorical variables were compared by Chi^2 ^test. Continuous variables, expressed as median and interquartile range (IQR), were compared by the Mann-Whitney U test. VAP incidence was expressed as the number of events per 1,000 invasive mechanical ventilation days of exposure and 95% confidence interval (CI).

Poisson regression was fitted to the data from D2 to D8 with the observed number of incident VAP as the dependent variable. Time was included as an independent variable with the number of invasive mechanical ventilation days at risk as offset in the model. The addition of a quadratic term for time was tested with the likelihood ratio test and conserved to take into account the non-linearity of incidence with time. Predictions of early-onset VAP incidence rates at D0 and D1 were based on the final model fitted. This model also estimated missed early-onset VAP occurring within 48 hours after admission. All tests were 2-tailed, p < 0.05 was considered significant. The data were analysed with Stata 8.0 software (Stats Corp. 2003. Stata Statistical Software: Release 8. College Station, TX: StataCorp LP).

## Results

### Population characteristics

A total of 20,640 patients were included in the surveillance in 11 ICUs over the study period. As a whole, 9,322 (45.2%) were newly hospitalized patients without immediate previous hospital stay, 9,657 (47.8%) patients did not receive antibiotics before admission, and 17,302 (83.8%) were exposed to mechanical ventilation on the first day of ICU stay.

Overall, data on 3,387 patients (37.6% women), accounting for 45,760 patient-days, were analyzed. A total of 367 (10.8%) patients developed VAP within the first 9 days of ICU stay. The main patient characteristics are described in Table 1. Median age was 54.3 years (IQR 40-69 years), and median Simplified Acute Physiology Score II was 44 (32-56). Totally, 914 (27.0%) were trauma patients. Hospital mortality was 21.7%.

**Table 1:** Characteristics of patients with or without ventilator-associated pneumonia (VAP) within 9 days of ICU admission in University of Lyon hospitals (France), January 2001-December 2009

Characteristics	VAP within 9 days (n = 367)	No VAP within 9 days (n = 3,020)	p-value
Categorical variable, n (%)			
Year of admission			< 0.001
2001-2003	95 (25.9)	1,202 (39.8)	
2004-2006	116 (31.6)	937 (31.0)	
2007-2009	156 (42.5)	881 (29.2)	
Gender, female	109 (29.7)	1,165 (38.6)	0.001
Neutrophil count < 500/mm^3^	23 (6.3)	189 (6.3)	1.0
Trauma	160 (43.6)	754 (25.0)	< 0.001
Diagnosis category			< 0.001
Medical	152 (41.5)	1,844 (61.1)	
Surgical	214 (58.5)	1,176 (38.9)	
Deceased in-hospital	75 (20.5)	656 (21.8)	0.6
Continuous variable, median (interquartile range)			
Age, years	51 (36-66)	55 (40-69)	0.002
Simplified Acute Physiology Score	47 (36-56)	44 (32-56)	0.011
Length of hospital stay, days	21 (12-33)	5 (3-12)	< 0.001
Length of intubation, days	15 (9-25)	2 (1-8)	< 0.001

### VAP incidence

**Figure 1:**
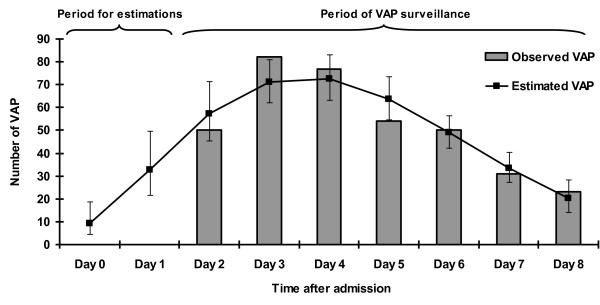
**Observed and estimated number (95% confidence interval) of VAP calculated by the Poisson regression model during the first 9 days of ICU stay in University of Lyon hospitals (France), January 2001-December 2009**.

The mean observed VAP incidence was 20.6 (95% CI 18.6-22.8) per 1,000 invasive mechanical ventilation days. Table 2 reports the VAP microbiological findings. Figure 1 depicts the distribution of observed and estimated VAP. The predicted cumulative incidence of early-onset VAP at D0 and D1 was 5.3 (95% CI 2.6-9.8) and 8.3 (95% CI 6.1-11.1) per 1,000 invasive mechanical ventilation days, respectively (Table 3). VAP incidence, estimated by the Poisson model, was 23.0 (95% CI 20.8-25.3) per 1,000 invasive mechanical ventilation days at D8. The total proportion of missed early-onset VAP during the first 48 hours of ICU stay was 11% (95% CI 9%-17%).

**Table 2:** Microbiological findings on patients who developed ventilator-associated pneumonia in ICUs of University of Lyon hospitals (France), January 2001-December 2009

Microorganism, n (%)	Day 2 (n = 50)	Day 3 (n = 82)	Day 4 (n = 77)	Day 5 (n = 54)	Day 6 (n = 31)	Day 7 (n = 23)	Day 8 (n = 23)
*Staphylococcus aureus*	18 (36)	35 (43)	28 (36)	23 (43)	19 (38)	8 (26)	6 (26)
*Streptococcus pneumoniae*	8 (16)	9 (11)	11 (14)	5 (9)	4 (8)	3 (10)	2 (9)
*Haemophilus influenzae*	10 (20)	14 (17)	16 (21)	14 (26)	9 (18)	4 (13)	3 (13)
*Pseudomonas aeruginosa*	3 (6)	1 (1)	5 (6)	5 (9)	2 (4)	3 (10)	5 (22)
Enterobacter	4 (8)	5 (6)	8 (10)	4 (7)	4 (8)	8 (26)	13 (39)
Others	18 (26)	24 (29)	17 (19)	16 (32)	10 (32)	4 (17)	2 (9)

**Table 3:** Observed and estimated ventilator-associated pneumonia (VAP) in ICUs of University of Lyon hospitals (France), January 2001-December 2009

	Period of VAP estimation		Period of VAP surveillance						
		
Characteristics	Day 0	Day 1	Day 2	Day 3	Day 4	Day 5	Day 6	Day 7	Day 8
Observed cumulative invasive mechanical ventilation days at risk	1,693.5	5,059.5	8,382.5	10,944.5	12,960.5	14,549.5	15,858.5	16,926.5	17,792.5
Observed number of VAP	-	-	50	82	77	54	50	31	23
Estimated number of VAP (95% CI)	9 (5-19)	33 (22-50)	57 (46-72)	71 (62-81)	73 (63-83)	64 (55-74)	49 (42-57)	33 (27-40)	20 (14-28)
Observed cumulative incidence of VAP (95% CI)	-	-	6.0 (4.5-7.8)	12.1 (10.1-14.3)	16.1 (14.1-18.4)	18.1 (16.0-20.4)	19.7 (17.6-22.0)	20.3 (18.3-22.6)	20.6 (18.6-22.8)
Estimated cumulative incidence of VAP (95% CI)	5.3 (2.6-9.8)	8.3 (6.1-11.1)	11.8 (9.7-14.3)	15.5 (13.3-18.0)	18.8 (16.5-21.2)	21.1 (18.8-23.6)	22.5 (20.2-24.9)	23.0 (20.1-25.4)	23.0 (20.8-25.3)

CI: confidence interval

## Discussion

The objective of the study was to estimate the incidence of early-onset VAP shortly after admission in ICU. The estimated incidence of early-onset VAP in ICUs within 48 hours after admission was 8.3 (95% CI 6.1-11.1) per 1,000 invasive mechanical ventilation days. It should be noted that these results cannot be generalized to ICU patient populations because patients were restricted to those at high risk of VAP with mechanical ventilation exposure and no antibiotic use at admission, which might overestimate the daily incidence. Therefore, this sample accounts for a population with high risk of VAP. Indeed, patients who receive antibiotics before ICU admission are at lower risk of VAP [18,19] or VAP within 48 hours after admission [13]. The same is true for previously-intubated patients whose VAP risk decreases over time [20].

To the best of our knowledge, data on the incidence of early-onset VAP are sparse. In a prospective study of 250 intubated ICU patients, 32 (12.8%) incurred VAP within 48 hours [13]; among them, 18 had VAP within 24 hours. However, that study did not provide incidence density, but focused on risk factors for pneumonia within 48 hours of tracheal intubation. The microorganism that they isolated most frequently was *Staphylococcus aureus *[13]; the same pattern was found for VAP in our study within 9 days. However, *Pseudomonas aeruginosa*, infrequent in our series, is a frequent etiological microorganism in VAP [21].

In early-onset VAP, we cannot totally exclude the occurrence of community-acquired pneumonia in the incubation period before admission or early aspiration-associated pneumonia at admission. VAP within 48 hours after hospitalization might be related to the acute period of mechanical ventilation, the severity of the underlying disease or both. Later infections could be associated with chronic exposure to mechanical ventilation and the evolution of the underlying disease. The distinction between acute and chronic risks of VAP might lead to specific preventive measures [19]. In early-onset VAP, the bacteria involved and the risk-factors for pneumonia might be mostly related to the reason for admission. On the other hand, late-onset VAP might be due more to the duration of exposure to invasive mechanical ventilation, quality of care or environmental ecology of the unit.

Our study has some limitations. First, no data were available concerning nasogastric tubes and aspiration, although patients with trauma could suffer aspiration pneumonia. Also, we were unable to control for this risk factor of pneumonia. However, the pathogens found most frequently were not Gram-negative bacilli, which did not suggest aspiration pneumonia. Second, the Glasgow coma scale, which can help to distinguish between VAP and aspiration pneumonia, was not available. Third, the external validity of the results was limited by initial population selection. However, this selection permitted us to emphasize the magnitude of the problem in patients with high risk of VAP.

The results need to be confirmed in prospective studies with data on early-onset VAP. Ideally, a large prospective investigation should be undertaken, including patients since the first day of admission, the precise time of exposure to mechanical ventilation as well as nasogastric tubes. Such a study, with microbiological and clinical data collection since the first day of admission, would permit differentiation between community- and hospital-acquired infections. Our results should encourage the surveillance of infection features shortly after ICU admission. In this setting, increased clinical monitoring and vigilance of early-onset VAP as well as early epidemiological surveillance of HAIs should be reinforced. Moreover, the identification of risk factors of early VAP might be helpful to improve clinical care and to prevent these infections.

## Conclusions

In summary, our study suggests possible underestimation of VAP incidence in ICUs. This finding should encourage clinicians to develop a strategy for the quick detection of early-onset VAP shortly after ICU admission. In such a setting, active, early surveillance of VAP features might improve clinical diagnosis and patient outcome. The evaluation of preventive measures against early-onset VAP, immediately after device exposure, should be encouraged.

## Competing interests

The authors declare that they have no competing interests.

## Authors' contributions

PV, TB, NV, and JMJ contributed to the study design and data analysis. PV drafted the initial manuscript. AL, BA, LA, DC, and CG contributed to the analysis and interpretation of data. All authors read, commented on and approved the final manuscript version. The Study Group contributed to the hospital-acquired infection surveillance network.

## Pre-publication history

The pre-publication history for this paper can be accessed here:

http://www.biomedcentral.com/1471-2334/11/236/prepub
